# Differential correlates of criticism versus emotional overinvolvement towards patients with schizophrenia living in halfway houses or with their families

**DOI:** 10.1007/s00127-023-02609-7

**Published:** 2024-01-31

**Authors:** Panagiotis Ferentinos, Stamatina Douki, Eleni Kourkouni, Dimitra Dragoumi, Nikolaos Smyrnis, Athanassios Douzenis

**Affiliations:** 1 2nd Department of Psychiatry, National and Kapodistrian University of Athens, “Attikon” University General Hospital, 1 Rimini Street, 124 62, Athens, Greece; 2 Department of Psychiatry, “Evangelismos” General Hospital, Athens, Greece; 3 Center for Clinical Epidemiology and Outcomes Research, Athens, Greece

**Keywords:** Criticism, Differential correlates, Emotional overinvolvement, Expressed emotion, Halfway houses, Schizophrenia

## Abstract

**Purpose:**

This study systematically searched for differential correlates of criticism vs. emotional overinvolvement (EOI) towards patients with schizophrenia in families and halfway houses, which have only incidentally been reported in previous research. Identified patterns were compared across settings.

**Methods:**

We included 40 inpatients with schizophrenia living in halfway houses and 40 outpatients living with their families and recorded the expressed emotion (EE) of 22 psychiatric nurses or 56 parents, respectively, through Five Minutes Speech Samples. Each nurse rated 1–12 inpatients and each inpatient was rated by 2–5 nurses. Each outpatient was rated by one or both parents. As EE ratings had a multilevel structure, weighted Spearman correlations of criticism and EOI with various patient- and caregiver-related characteristics were calculated and compared with Meng’s z-test.

**Results:**

Criticism was weakly negatively correlated with EOI in nurses but negligibly in parents. Distinct patterns of significant differential correlates arose across settings. Outpatients’ aggressive behavior and parents’ related burden were mainly associated with higher criticism. Inpatients’ symptoms (agitation/aggression, negative and other psychotic symptoms) and nurses’ burnout (Depersonalization) were mainly associated with lower EOI. Inpatients’ perceived criticism and outpatients’ previous suicide attempts were equally associated with higher criticism and lower EOI (mirror correlations). Finally, various inpatient attributes (older age, chronicity, unemployment and smoking) triggered higher EOI only. Inpatients’ age, psychopathology (esp. agitation/aggression and negative symptoms) and perceived criticism survived adjustment for multiple comparisons.

**Conclusion:**

Our findings suggest setting-specific pathogenetic pathways of criticism and EOI and might help customize psychoeducational interventions to staff and families.

**Supplementary Information:**

The online version contains supplementary material available at 10.1007/s00127-023-02609-7.

## Introduction

The family environment is recognized as a major modifiable predictor of the course of schizophrenia along with stressful life events, reduced compliance to treatment, substance misuse and poor premorbid adjustment [1–3]. The family environment is associated with the emergence of relapses through ‘‘expressed emotion’’ (EE) [4], a construct introduced in the 1950s to describe affective attitudes and behaviors determining the quality of intrafamilial emotional communication. It includes five dimensions [5] as traditionally recorded with the gold standard tool, Camberwell Family Interview (CFI) [1]: three negative dimensions, which have drawn much greater attention and are used in scoring EE, i.e., criticism (critical, resenting or disapproving comments about the patient’s behavior), hostility (extreme form of criticism and rejection, often highly correlated with criticism), and emotional overinvolvement (EOI, evidenced by exaggerated emotional responses, over-intrusive, over-protective or self-sacrificing behavior, and over-identification with the patient), as well as two positive dimensions, i.e., warmth (empathy, concern and interest for the patient, usually negatively correlated with criticism and positively correlated with EOI) and positive remarks (praising, appreciating or approving comments), which do not count in scoring EE but are increasingly attracting attention in more recent studies. Apart from the time-consuming CFI, ΕΕ is assessed with the far briefer Five Minutes Speech Sample, measuring only criticism and EOI, i.e., the two key EE components, and several self-report questionnaires for caregivers [6, 7], such as the Patient Rejection Scale [8], the Family Attitude Scale [9] and the Family Questionnaire [10]. Meta-analyses of various prospective studies documented the negative impact of high EE, particularly criticism and to a far lesser extent EOI, on clinical outcomes in family settings [11, 12] while high warmth, particularly in the absence of EOI, acts protectively [13].

During the last three decades, research on EE in schizophrenia has extended to the staff (usually mental health nurses) of psychosocial rehabilitation services. Among them, supported housing facilities in the community, a cornerstone of deinstitutionalization, are comparable to the patient’s family environment. However, as staff may emotionally invest less in patients than family relatives, high EE in staff-patient studies almost always arises from criticism rather than EOI, which seems less relevant in these settings [14]. Tools to assess staff EE are generally the same as in families but setting-specific tools, such as the Nurse Attitude Scale [15], are increasingly used. Staff EE or its components have also been associated with patient outcomes though weakly, inconsistently and in much fewer prospective studies [16–19].

Given the importance of EE for clinical outcomes, various studies have investigated patient- and caregiver-related characteristics associated with EE in family and staff-patient settings [14, 20]. Although studies rarely report correlations of criticism with EOI, it has been suggested that they are relatively independent in family relatives and often have different correlates [21]. Specifically focusing on differential correlates of criticism vs. EOI may highlight specific pathways giving rise to each EE dimension. For example, patients’ perceived criticism is often recognized as a differential predictor of criticism but not EOI in family relatives [22, 23] but also in staff [24, 25]. However, differential correlates of criticism vs. EOI have not been systematically investigated in both families and staff. The only study that explicitly had this specific aim included patients with first episode psychosis and identified duration of untreated psychosis and family relatives’ distress as differential correlates of criticism vs. EOI [21]. Various other studies only incidentally reported contrasting patterns of correlations of criticism and EOI with patients’ demographics (gender [26], age [27], employment [27, 28]), clinical characteristics (disease duration [29], previous hospitalizations or psychotic episodes [28, 30, 31], history of aggressive behavior [32]), current psychopathology or aggressive behavior [33–36], and PC [22, 23, 25] or caregivers’ demographics (parents’ gender and employment [30, 36], nurses’ age, education and work experience [29, 37]), causal attributions/ illness perceptions [24, 38–40], distress [32], coping strategies [32, 35], duration of contact with patients [33] and personality factors [29]. However, studies above have not documented that differences in correlations of various features with criticism and EOI were statistically significant as this was not their aim. Furthermore, several features have scarcely been investigated as differential correlates, such as parents’ psychiatric history (given that parents with affective disorders display higher criticism [41]) and patients’ history of suicide attempts or smoking status, while others need further research. For example, caregiver burden, as a global score, was positively associated with both criticism and EOI [21, 42, 43] but its domain-specific dimensions have not been studied with regard to criticism and EOI; emotional burnout was positively associated with criticism and negatively with positive remarks (i.e., not EOI) as assessed with the NAS [15, 44] but this finding needs to be replicated with other EE tools measuring criticism and EOI.

Finally, it is unknown whether patterns of differential correlates are similar among families and hostels. EE correlates can only indirectly be compared between the two settings since studies including patients in both settings are unfortunately absent. However, indirect comparisons can suffer from many biases. EE and other measures used, scoring algorithms and procedures followed as well as EE predictors investigated can be different. Comparing patterns across settings might uncover setting-specific pathogenetic pathways of criticism and EOI and help customize psychoeducational interventions to staff and families.

This study included patients with schizophrenia living in halfway houses or with their families and recorded the EE of the caring staff or parents, respectively, towards the patients. It aimed to systematically investigate differential correlates of criticism vs. EOI in each setting among various patient- and caregiver-related characteristics, including most of those with some previous supporting evidence, outlined above, as well as few understudied ones, and directly compare identified patterns across settings. We hypothesized that these features would display significantly different correlations with criticism and EOI and that patterns of significant differential correlates would be similar across settings.

## Materials and methods

### Participants

A convenient sample of 80 patients of both sexes with a DSM-5-based diagnosis of schizophrenia, aged 18–65 years, was recruited during a two-year period; 40 inpatients lived in four transitional halfway houses (psychiatric hostels) for at least 3 months and 40 patients lived with their families and were followed-up in two general hospital outpatient clinics. All patients had to be on antipsychotic medication, free of relapse and in no need for psychiatric hospitalization during the last 3 months. Exclusion criteria for inpatients’ admission into the hostels were intellectual disability, history of alcoholism or drug use in the last 6 months and current severe medical conditions (e.g., neurological degenerative diseases, brain lesions); these were also applied in recruiting both inpatients and outpatients. Patients’ clinicodemographic characteristics were recorded.

In addition, twenty-two nurses working in the four halfway houses and caring for the 40 inpatients and 56 parents of the 40 outpatients also participated in the study as raters of their EE towards patients. An additional exclusion criterion for raters was lifetime diagnosis of psychotic disorder.

Both patients and raters were ensured about the anonymity and confidentiality of all data requested and provided written informed consent before participation in the study. The research protocol followed the principles of the Helsinki Declaration and was approved by the Research Ethics Committees of all mental health facilities involved.

### Measurements

Patients of both groups went through the following evaluations:

- *Brief Psychiatric Rating Scale (BPRS)* BPRS originally included 16 interviewer-rated items assessing the intensity of symptoms of schizophrenia [45]. The most commonly used 18-item version (with the addition of excitement and disorientation in 1966) has a five-factor structure, including Thinking disorder, Withdrawal, Anxiety/Depression, Hostility/Suspicion, and Activity factors [46]. The Greek version had a Cronbach’s α = 0.80 for the total scale [47].
- *Perceived Criticism (PC)* The PC instrument was introduced to measure perceived criticism in a sample of depressed patients and their spouses [48] but has since been used with several other populations, including patients with schizophrenia [49, 50]. It consists of only one self-rated question on a 10-point Likert scale: “How critical do you feel hostel nurses/your parents have been of you overall in the last month?”.

Staff nurses caring for inpatients in psychiatric hostels underwent the following assessment:

- *Maslach Burnout Inventory (MBI)* The scale of professional burnout was designed by Maslach and Jackson in 1981 and amended in 1996 [51, 52]. It consists of 22 self-evaluation items scored 0–6 and explores the feelings and attitudes of professionals in their work. The scale consists of three subscales measuring Emotional Exhaustion (9 items), Depersonalization (5 items) and Personal Achievements (8 items); higher scores in the first two subscales and lower ones in the third suggest higher burnout. The Greek version of the MBI had satisfactory psychometric properties in a sample of nurses [53].

The parents of outpatients living with their families were evaluated as follows:

- *Family Burden Scale (FBS)* The FBS was designed by Madianos and Economou in 1993 and in 2004 it was amended to its final form [54], which displayed satisfactory validity and reliability (Cronbach’s α = 0.85). It is a structured interview for the relatives of patients with schizophrenia and explores the burden of the mental illness on them in the last 6 months. The scale consists of 4 subscales with 23 questions in total rated 0–2: Financial Burden (5 items), Impact on Daily Activities and Social Life (8 items), Aggressive Behavior (4 items) and Impact on Health (6 items). The first three subscales indicate the objective burden, while the fourth indicates the subjective burden. The total score ranges from 0 to 46.

Finally, both staff nurses and outpatients’ parents participated in the following procedures:

- *Five Minutes Speech Sample (FMSS)* The FMSS [55] is a tool for measuring EE. In relation to CFI, the standard assessment tool of EE, the FMSS is easier to use, needs far less time to administer, and requires shorter training of the interviewer. It can also be used even when the investigator does not know the patient very well. Each rater/caregiver is asked to talk continuously for 5 min about each patient (in his/her absence) and the interview is audiotaped. All recorded 5 min interviews are then scored according to specific rules based on the assessment of: (a) the initial statement (content, voice tone), (b) the quality of the patient-rater relationship, (c) the number of negative or positive comments and d) the display or report of specific behaviors during the interview (see Supplementary Methods for details on scoring). Every 5 min speech sample is eventually characterized as high, borderline or low on criticism and EOI; combined classifications also arise (e.g., ‘high critical’, ‘high EOI’, ‘high critical + EOI’). Although FMSS is considered less sensitive than the CFI in detecting high-EE individuals [6], its validity in predicting relapse of schizophrenia has been established [56]. FMSS interviews were scored by a trained author (S.D.) and acceptable inter-rater agreement with another trained author (P.F.) was recorded in 20 interviews (criticism: 95% agreement on 6 low- and 13 high-criticism ratings, kappa = 0.89; EOI: 95% agreement on 7 low- and 12 high-EOI ratings, kappa = 0.89).

### Statistical analysis

The distribution of all variables was explored with descriptive statistics. Normality was checked with the Shapiro–Wilk test and graphically with histograms and QQ-plots. Reliability (internal consistency) of the returned questionnaires was evaluated with Cronbach’s alpha. Differences in characteristics of participants (i.e., patients and nurses or parents) and EE outcomes between the two groups were evaluated with chi-square, Fisher’s exact, t-test or Mann–Whitney tests, as appropriate. As frequencies of low FMSS-Criticism/EOI categories were very small, they were collapsed with borderline FMSS-Criticism/EOI categories in downstream analyses.

**Fig. 1:**
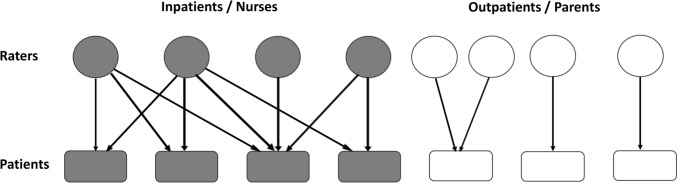
Multilevel structure of expressed emotion ratings made by raters (circles) for patients (rectangles) in the two settings (grey, inpatients/nurses; white, outpatients / parents)

EE ratings derived from FMSS interviews were a multi-level dataset of observations. In particular, each inpatient received ratings from various nurses and each nurse rated several inpatients (‘crossed levels’) while parents’ ratings were nested within outpatients (Fig. 1). Pairwise Spearman correlations between criticism (high and borderline/low FMSS-Criticism) and EOI (high and borderline/low FMSS-EOI) were calculated in each patient group and in the total sample. Correlations were calculated as either unweighted or weighted, using ‘wCorr’ and ‘weights’ (‘wtd.cor’ function) R packages and either of two sampling weights, 1/Npat (Npat = number of ratings made for each patient) or 1/Nrat (Nrat = number of ratings each rater made), so as to take into account the hierarchical/ multilevel structure of EE ratings. Weights were based on the inverse of the probability of each patient’s or each rater’s ratings to be included in the total sample of ratings; respective probabilities were proportional to Npat or Nrat. Furthermore, we calculated weighted Spearman correlations of criticism and EOI with caregiver-related features (weighted by 1/Nrat) and patient-related features (weighted by 1/Npat) in each patient group. Therefore, for example, patients rated by more nurses (i.e., with higher Npat) were downweighted when their BPRS was correlated with their FMSS-Criticism ratings while nurses rating more patients (i.e., with higher Nrat) were downweighted when their MBI was correlated with their FMSS-EOI ratings.

To identify differential correlates of criticism vs. EOI, the weighted correlations of each feature with FMSS-Criticism and FMSS-EOI in each group were compared with Meng’s z-test [57], an improved version of William’s and Steiger’s tests, using ‘cocor’ R package. This is appropriate for comparing correlation coefficients which are correlated (i.e., in the same sample) and overlapping (i.e., sharing a variable), e.g., comparing correlations A-B vs. A-C, where A is a rater or patient feature, B is FMSS-Criticism and C is FMSS-EOI. Meng’s test produces a z-score which depends on correlations A-B, A-C and B-C and its absolute value increases with sample size.

Statistical analysis was conducted in STATA MP v17 and R 4.1.2. Given the exploratory nature of the study, the level of statistical significance was set to p < 0.05 (two-tailed). However, the most robust findings were identified by adjusting for multiple comparisons (24 in hostels and 26 in families) using a strict corrected p < 0.001 (two-tailed).

## Results

### Sample descriptives, univariate comparisons and correlations

#### Patients (inpatients-outpatients)

Age and disease duration did not significantly deviate from normality while previous hospitalizations were not normally distributed. Inpatients were older and less well educated than outpatients and had a longer disease duration as well as more hospitalizations (Table 1).

**Table 1:** Description of patient sample (40 inpatients, 40 outpatients) and basic demographics for raters (22 nurses, 56 parents)

	Inpatients (N = 40)	Outpatients (N = 40)	p-value
Gender (Male)	27 (67.5%)	22 (55.0%)	0.251^a^
Age (years)	48.6 ± 9.3	40.1 ± 7.8	** < 0.001**^c^
Family status			1.000^b^
Single	34 (85.0%)	33 (82.5%)	
Married	1 (2.5%)	2 (5.0%)	
Divorced/widowed	5 (12.5%)	5 (12.5%)	
Education			**0.002**^a^
Primary/high school	33 (82.5%)	20 (50.0%)	
University or higher	7 (17.5%)	20 (50.0%)	
Employment			0.762^b^
Employed	7 (17.5%)	10 (25.0%)	
Unemployed	30(75.0%)	27 (67.5%)	
Retired	3 (7.5%)	3 (7.5%)	
Smoking	28 (70.0%)	24 (60.0%)	0.348^a^
History of violent behavior	14 (35.0%)	10 (25.0%)	0.329^a^
History of suicide attempts	3 (7.5%)	3 (7.5%)	1.000^b^
Duration of disease (years)	18.2 ± 10.9	13.4 ± 7.2	**0.025**^c^
No of hospitalizations	2 (2–4)	2 (1–3)	**0.012**^d^
BPRS Thinking disorder	5 (4–7.5)	6.5 (5–9.5)	0.190^d^
BPRS Withdrawal	6 (4–8)	8.5 (6–11.5)	**0.015**^d^
BPRS Anxiety/Depression	7.5 (6–9.5)	9 (6.5–12)	0.162^d^
BPRS Hostility/Suspicion	4 (3–5.5)	4 (3–6.5)	0.964^d^
BPRS Activity	3 (3–4)	3 (3–4.5)	0.345^d^
BPRS Total	28 (22.5–37)	33.5 (28.5–40.5)	**0.027**^d^
Perceived Criticism	3.7 ± 2.4	5.80 ± 2.66	** < 0.001**^c^
	**Nurses (N = 22)**	**Parents (N = 56)**	
Gender (Male)	6 (27.3%)	24 (42.9%)	0.203^a^
Age (years)	40.0 ± 7.2	68.0 ± 8.6	** < 0.001**^c^
Education			0.001^b^
Primary school	0 (0.0%)	20 (35.7%)	
High school	8 (36.4%)	19 (33.9%)	
University or higher	14 (63.6%)	17 (30.4%)	

Bold p < 0.05

N(%) or median(IQR) or mean ± SD are presented

*BPRS* Brief Psychiatric Rating Scale

^a^Chi-square

^b^Fisher's exact

^c^t-test

^d^Mann-Whitney

All patient questionnaires had adequate reliability (Cronbach’s α > 0.7) (Suppl. Table 1). PC was approximately normally distributed while BPRS total and subscales were positively skewed. Outpatients had significantly higher scores in BPRS Withdrawal, BPRS Total and PC than inpatients (Table 1). However, both patient groups were overall remitted [58], since BPRS Total scores around 30 approximately correspond to a score of 3 (‘mildly ill’) on the Clinical Global Impression- Severity scale [59].

#### Raters (nurses—parents)

Demographics for nurses and parents are presented in Table 1 and in further detail in Suppl. Table 2. Nurses were significantly younger than parents and had a higher level of education. Seven (12.5%) parents had a lifetime psychiatric history of depression. All raters’ questionnaires (MBI, FBS) had adequate reliability (Suppl. Table 1). Overall, nurses scored moderate on MBI Personal Achievements and low on other MBI subscales [51] while parents scored low on FBS total, with 12 (21.4%) scoring > 24 [54].

#### EE ratings

Each of the 22 nurses was involved in 1–12 EE ratings (FMSS interviews); each of the 40 inpatients was rated by 2–5 nurses. A total of 155 ratings were performed by nurses on inpatients. All 56 parents rated their offspring; each of the 40 outpatients was rated by one or both parents (Fig. 1).

There were no significant differences among groups in FMSS-Criticism and FMSS-EOI (Table 2). However, a marginally significant difference in the distribution of combined EE categories (mainly driven by the ‘borderline critical’ category) but not binary EE categories (high vs. low EE) was detected among groups.

**Table 2:** Comparison of Expressed Emotion (EE) outcomes between nurses/inpatients and parents/outpatients

Five Minutes Speech Sample (FMSS) outcomes	Nurses^a^	Parents^b^	p-value
Criticism			0.310^†^
High	84 (54.2%)	33 (58.9%)	
Borderline	65 (41.9%)	23 (41.1%)	
Low	6 (3.9%)	0 (0.0%)	
Emotional Overinvolvement (EOI)			0.940^**††**^
High	72 (46.5%)	27 (48.2%)	
Borderline	65 (41.9%)	22 (39.3%)	
Low	18 (11.6%)	7 (12.5%)	
EE categories (n = 7)			**0.049**^§^
High critical	54 (34.8%)	17 (30.4%)	
High EOI	42 (27.1%)	11 (19.6%)	
High critical + EOI	30 (19.4%)	16 (28.6%)	
Borderline critical	1 (0.6%)	4 (7.1%)	
Borderline EOI	0 (0.0%)	0 (0.0%)	
Borderline critical + EOI	28 (18.1%)	8 (14.3%)	
Low critical + EOI	0 (0.0%)	0 (0.0%)	
EE Categories (n = 2)			0.659
High EE	126 (81.3%)	44 (78.6%)	
Low EE	29 (18.7%)	12 (21.4%)	

Bold p < 0.05

N(%) are presented. Chi-square or Fisher’s exact tests were used as appropriate

^a^Data come from 155 nurses’ ratings (FMSS interviews); each of the 22 nurses was involved in 1–12 ratings; each of the 40 inpatients was rated by 2–5 nurses

^b^All 56 parents were involved in an FMSS interview for their offspring; each of the 40 outpatients was rated by one or both parents

^†^p = 0.541 for high vs. borderline/low FMSS-Criticism

^††^p = 0.821 for high vs. borderline/low FMSS-EOI

^§^p = 0.405 for four FMSS categories (high critical, high EOI, high critical + EOI, borderline/low critical + EOI)

As shown in Table 3, FMSS-Criticism was significantly weakly negatively correlated with FMSS-EOI in nurses but negligibly in parents. Weighted correlations followed the same pattern.

**Table 3:** Correlations of FMSS-Criticism with FMSS-EOI

	FMSS-Criticism (high vs. borderline/low)		
	Unweighted	Weighted by 1/Npat	Weighted by 1/Nrat
FMSS-EOI (high vs. borderline/low)	**−** **0.170, 0.013**	**− 0.142, 0.039**	− 0.062, 0.372
	**− 0.234, 0.003**	**− 0.238, 0.003**	**− 0.244, 0.002**
	0.007, 0.960	− 0.052, 0.705	0.006, 0.962

Bold, p < 0.05

Unweighted and weighted Spearman correlations are presented (rho, p-value). Weighted correlations have been calculated using two weights (1/Npat, 1/Nrat): Npat = number of ratings made for each patient; Nrat = number of ratings each rater made

Upper line = total sample (N = 211 ratings), middle line = inpatients rated by nurses (N = 155 ratings), lower line = outpatients rated by their parents (N = 56 ratings)

*FMSS* Five Minutes Speech Sample

### Differential correlates of criticism vs. EOI outcomes in each setting

Significant differential correlates of criticism vs. EOI were identified in each setting with Meng’s test (Table 4A, B). In hostels, nurses’ MBI Depersonalization and inpatients’ BPRS Thinking disorder, Withdrawal, Hostility/Suspicion, Activity and Total were weakly to moderately negatively associated with EOI, with much weaker positive associations with criticism; BPRS Activity had the strongest positive association with criticism. PC had a weak negative association with EOI and a weak positive association with criticism of similar size (mirror correlations). Inpatients’ age, unemployed status, smoking and disease duration were weakly to moderately positively associated with EOI, with much weaker negative associations with criticism; inpatients’ female gender (at trend level) had a weak positive association with EOI and a weak negative association with criticism of similar size (mirror correlations).

In families, parents’ FBS Aggressive Behavior and outpatients’ history of aggressive/violent behavior (at trend level) were weakly to moderately positively associated with criticism, with much weaker negative associations with EOI; previous suicide attempts had a weak positive association with criticism and a weak negative association with EOI of similar size (mirror correlations).

After adjusting for multiple comparisons (50 tests in both groups), the only significant (p < 0.001) differential correlates of criticism vs. EOI were identified in hostels and included patients’ age, BPRS Withdrawal, Activity, Total and PC.

### Comparison of patterns across settings: sensitivity analysis

The patterns of significant differential correlates were not directly comparable across settings as Meng’s z-score is sample size dependent and the number of ratings performed in the two settings was quite different (155 vs. 56). Therefore, as a sensitivity analysis, we recalculated Meng’s tests in hostels assuming that they were based on 56 rather than 155 ratings and keeping all other parameters unchanged (Table 4A, last column). Differential correlates now identified as significant (p < 0.05) were 5/24 in hostels compared to 2/26 in families. The two proportions were not significantly different (Fisher’s exact, p = 0.24).

**Table 4:** Differential correlates of criticism vs. EOI in inpatients (A) and outpatients (B)

(A) Inpatients (155 ratings)	FMSS—criticism	FMSS—EOI	Meng’s test (z)	p-value	p-value scaled to 56 ratings
Nurse features^a^					
Female gender	0.210**	0.255**	− 0.368	0.713	0.828
Age	0.211**	0.247**	− 0.294	0.769	0.862
Family status (married vs. single)	0.074	0.094	− 0.157	0.875	0.926
Education (higher vs. secondary)	− 0.089	− 0.185*	0.762	0.446	0.653
Work experience (> 11 years vs. lower)	0.028	− 0.002	0.235	0.815	0.890
MBI Emotional exhaustion	0.056	− 0.062	0.923	0.356	0.586
MBI Personal achievements	− 0.002	0.095	− 0.760	0.447	0.654
MBI Depersonalization	0.060	− 0.215**	2.167	0.030*	0.201
Patient features^b^					
Female gender	− 0.135	0.110	− 1.924	0.054	0.256
Age	− 0.138	0.365**	− 4.031	0.0001**	0.017*
Education (university or higher vs. lower)	− 0.135	0.071	− 1.618	0.106	0.339
Unemployed (vs. employed/retired)	− 0.099	0.213**	− 2.460	0.014*	0.146
Smoking	− 0.076	0.238**	− 2.483	0.013*	0.143
Disease duration	− 0.079	0.216**	− 2.328	0.020*	0.169
No of previous hospitalizations	− 0.045	0.072	− 0.917	0.359	0.588
History of violent behaviour	− 0.005	− 0.036	0.243	0.808	0.886
History of suicide attempts	0.025	− 0.072	0.761	0.447	0.653
BPRS Thinking disorder	0.016	− 0.327**	2.758	0.006**	0.103
BPRS Withdrawal	0.113	− 0.421**	4.334	< 0.0001**	0.010*
BPRS Anxiety/Depression	0.067	− 0.127	1.523	0.128	0.368
BPRS Hostility/Suspicion	0.082	− 0.180*	2.062	0.039*	0.223
BPRS Activity	0.166*	− 0.326**	3.916	0.0001**	0.021*
BPRS Total	0.142	− 0.439**	4.724	< 0.0001**	0.005**
Perceived Criticism	0.257**	− 0.231**	3.861	0.0001**	0.023*

(B) Outpatients (56 ratings)	FMSS—criticism	FMSS–EOI	Meng’s test (z)	p-value
Parent features^a^				
Female gender	− 0.063	0.258	− 1.674	0.094
Age	0.139	0.152	− 0.068	0.946
Education (higher vs. primary/secondary)	0.078	0.062	0.083	0.934
Currently employed	− 0.074	0.015	− 0.460	0.646
Psychiatric history	− 0.014	0.068	− 0.424	0.672
FBS Financial burden	0.059	0.117	− 0.301	0.763
FBS Impact on activities/social life	0.246	0.052	1.020	0.308
FBS Aggressive behavior	0.366**	− 0.069	2.300	0.022*
FBS Impact on health	0.094	− 0.122	1.117	0.264
FBS Total	0.253	0.001	1.320	0.187
Patient features^b^				
Female gender	0.078	0.201	− 0.627	0.531
Age	− 0.057	0.119	− 0.885	0.376
Education (university or higher vs. lower)	0.155	0.300*	− 0.759	0.448
Unemployed (vs. employed/retired)	0.069	− 0.053	0.613	0.540
Smoking	0.000	− 0.051	0.256	0.798
Disease duration	0.001	0.035	− 0.171	0.864
No of previous hospitalizations	0.057	− 0.027	0.422	0.673
History of violent behaviour	0.268*	− 0.115	1.940	0.052
History of suicide attempts	0.221	− 0.190	2.073	0.038*
BPRS Thinking disorder	0.062	− 0.020	0.412	0.681
BPRS Withdrawal	0.089	0.035	0.272	0.786
BPRS Anxiety/Depression	− 0.055	− 0.009	− 0.231	0.817
BPRS Hostility/Suspicion	0.018	− 0.141	0.801	0.423
BPRS Activity	0.091	− 0.052	0.718	0.473
BPRS Total	0.048	− 0.023	0.357	0.722
Perceived Criticism	0.163	− 0.046	1.053	0.292

Bold, significant after adjusting for multiple comparisons (p < 0.001 two-tailed)

Weighted Spearman correlations of each feature with FMSS—Criticism and FMSS—EOI are presented

*BPRS* Brief Psychiatric Rating Scale, *FBS* Family Burden Scale, *MBI* Maslach Burnout Inventory

^a^Weighted by 1/Nrat (Nrat = number of ratings each rater made)

^b^Weighted by 1/Npat (Npat = number of ratings made for each patient)

^*^p < 0.05

**p < 0.01

## Discussion

This study adds to a large literature on EE and its correlates in families of patients with schizophrenia as well as a smaller, more recent literature in staff-patient settings. It is novel in simultaneously recording with FMSS interviews and directly comparing EE across parents and professional caregivers. We systematically searched in each setting for differential correlates of criticism vs. EOI among various patient- and caregiver-related features, including most of those with previous supporting evidence and few understudied ones, and compared identified patterns across settings.

The EE rate in parents (Table 2) is at the upper end of meta-analytical reports but criticism and EOI rates are less than 1 S.D. higher than the means of previous studies in families [12, 60]. On the other hand, the EE rate in nurses was unexpectedly high, since rates are typically lower than 40% in staff-patient studies, with negligible rates of EOI [14]. Rates of criticism were, expectedly, larger than EOI in both settings. Cultural variation [61], especially regarding the EOI construct [62], individual characteristics of families and hostels and author’s scoring style might provide explanation for these inflated EE rates.

In our study, criticism and EOI were found uncorrelated in parents but weakly negatively correlated in nurses (Table 3). FMSS-EOI is known to capture part of CFI’s warmth dimension (concern and interest for patients) through positive comments [5, 6], suggesting that high criticism in nurses was associated with disengagement from patients. In fact, this explains why nurse ratings were more often either ‘high critical’ or ‘high EOI’ and less often ‘high critical + EOI’ than parent ratings (Table 2). Therefore, differential correlates of criticism vs. EOI were expected to be more relevant for hostels. A much richer pattern of significant differential correlates was indeed identified in inpatients, but this is partly explained by many more ratings performed in this group, lending higher power in Meng’s test. After adjusting for the different number of ratings between groups in a sensitivity analysis, inpatients still displayed more differential correlates but not to a significant extent.

Previous supporting evidence for differential correlates of criticism vs. EOI is incidentally reported and inconsistent in both families and staff-patient settings. In nurse-inpatient settings, patients’ overall psychopathology (particularly, negative symptoms/poor social functioning and behavioral disturbance/aggressive- agitated behavior) [24, 29, 33, 34, 37] and patients’ PC [24, 25] were positively associated with criticism and/or negatively with EOI, in line with our findings. We also identified positive associations of inpatients’ age and disease duration with EOI only; in previous studies, age was positively associated with criticism and EOI [29] and disease duration with criticism only [29]. Furthermore, inpatients’ unemployed status and smoking were associated with higher EOI only, while female gender (at trend level) had mirror correlations with criticism and EOI; these findings were not previously reported. Regarding nurse characteristics, in our study MBI Depersonalization was negatively associated with EOI only, a finding not previously reported; MBI Depersonalization and Emotional Exhaustion were previously associated with higher criticism, as recorded with the NAS, and fewer positive remarks (i.e., not EOI) while personal achievements followed the reverse pattern of associations [44]. In our study, nurses’ female gender and age were positively associated with both criticism and EOI (i.e., without differential effects) while higher education mainly with lower EOI; earlier studies reported associations of nurses’ older age, lower education and longer work experience with higher criticism only [29, 37] but many others found no significant EE associations with staff demographics [16, 17, 33].

On the other hand, in families, outpatients’ psychopathology and PC did not have significant differential effects on criticism vs. EOI in our study; yet, history of patients’ aggressive behavior (at trend level) and parents’ related burden were positively associated with criticism only. Previous studies recorded positive associations of patients’ current or past aggression with criticism only [32, 35] and negative ones with EOI [36], positive associations of patients’ depression/ anxiety with both criticism and EOI [28, 36], but most studies reported no EE associations with patients’ current psychotic symptoms [21, 40, 49]. Previous suicide attempts were equally associated (i.e., had mirror correlations) with higher criticism and lower EOI in our study, a finding not previously reported; an earlier study reported non-significant associations in the same direction [63]. Previous evidence on other characteristics not identified as differential correlates in our study included positive associations of patients’ PC with criticism only [22, 23] or both criticism and EOI [43], and positive associations of previous hospitalizations or psychotic episodes with criticism only [28, 31, 36] or EOI only [30]. Patients’ age was positively or negatively associated with criticism and negatively with EOI [27, 42], male patients received higher criticism only [26], while patients’ unemployment was associated with higher criticism only [28] or higher EOI only [27]. Regarding parents’ characteristics, global burden was most often positively associated with both criticism and EOI in earlier studies [21, 42, 43], while female [27, 30, 35, 36] and unemployed [30] parents scored higher on EOI only.

Therefore, families and hostels had quite distinct patterns of differential correlates of criticism vs. EOI. The interpretation of our findings may help formulate hypotheses about specific pathogenetic pathways of criticism and EOI in the two settings. First, patients’ aggressive/ agitated behavior (current or past) was associated with higher criticism in both groups. However, this association was stronger in families, probably because outpatients were younger and had more severe symptoms than inpatients while their parents were older and less well educated than nurses. Higher criticism may suggest modifiable causal attributions of schizophrenia to personal, internal and controllable factors [24, 38–40]. Second, in hostels patients’ symptoms (agitation/ aggression, negative and other psychotic symptoms) were mainly associated with lower EOI, i.e., disengagement from patients. EOI is lower in hostels as staff usually emotionally invest less in patients than family relatives [14]. Third, the impact of patients’ symptoms on caregivers followed the same pattern, i.e., parents’ aggression-related burden was mainly associated with higher criticism while nurses’ burnout (Depersonalization) with lower EOI. Fourth, two mirror correlations were identified. Patients’ PC in hostels and previous suicide attempts in families were equally associated with both higher criticism and lower EOI, i.e., a maximal and multi-faceted EE response. In both settings, the direction of causality between criticism/EOI and patients’ behaviors/ attitudes cannot be deduced, and circular causation is highly probable. Finally, various inpatient attributes (i.e., older age, chronicity, unemployment and smoking) triggered higher EOI only, reflecting nurse’s concern for certain patient categories. Overall, inpatients’ age, psychopathology (esp. agitation/ aggression and negative symptoms) and PC were our most robust differential correlates. To the best of our knowledge, our findings regarding nurses’ MBI Depersonalization, inpatients’ age, unemployment, smoking and disease duration, parents’ FBS Aggression and outpatients’ previous suicide attempts were not previously reported.

The aforementioned hypotheses might help customize the objectives and therapeutic components of psychoeducational interventions to families [64, 65] or professional caregivers [66, 67], aspiring to enhance their caregiving capacity. Psychoeducation of families should aim to increase knowledge about schizophrenia or associated behaviors (aggression, suicidality), modify their causal attributions, increase competency to face emergencies and help endorse more adaptive coping strategies to alleviate burden or avoid disengagement [68]. Psychoeducation to staff should aim to improve understanding of patients’ aggression, criticism or negative symptoms, encourage tolerance and a non-critical attitude, inspire therapeutic dynamism and optimism, motivate interventions targeting patients’ health-related problems (e.g., smoking, inactivity, aging) or psychosocial status (e.g., vocational or social skills training) in order to avoid burnout and disengagement [69].

### Strengths and limitations

Most previous EE studies used patient-caregiver dyads after arbitrarily selecting one ‘primary’ caregiver for each patient in order to simplify statistical analyses, yet unavoidably introducing bias. Instead, we have allowed each patient to be rated by 2–5 nurses or 1–2 parents while each nurse rated 1–12 patients; therefore, another strength of this study was our recruitment scheme (‘one patient by many raters, one rater for many patients’), which provides more valid and less biased EE ratings.

Limitations of our study include: (a) a relatively small sample size for both groups; (b) much fewer EE ratings in outpatients (56 vs. 155 in inpatients); therefore, the outpatient group had less power to detect significant associations and differential correlates; however, our sensitivity analysis adjusted for the different number of EE ratings between groups, making results comparable among them; (c) additional patient (duration of untreated psychosis) or caregiver (distress, causal attributions/ illness perceptions, coping strategies, personality profile) characteristics with previous supporting evidence as differential correlates might have also been investigated. Finally, duration of contact with patients (e.g., time since inpatients’ admission to hostels), previously associated with both EE components [29, 33], is a potential confounder not controlled in our study.

## Conclusion

In this study, we systematically searched in families and psychiatric hostels for differential correlates of criticism vs. EOI towards outpatients or inpatients, respectively, with schizophrenia. We found that some patient symptoms and their impact on caregivers were mainly associated with distinct EE dimensions in each setting (outpatients’ aggression and parents’ related burden with higher criticism; inpatients’ aggression, psychotic symptoms and nurses’ burnout with lower EOI) while inpatients’ perceived criticism and outpatients’ previous suicide attempts were equally associated with higher criticism and lower EOI. Certain patient attributes (older age, chronicity, unemployment and smoking) mainly triggered higher EOI in hostels. Our findings suggest setting-specific pathogenetic pathways of criticism and EOI and might inform psychoeducational interventions to staff and families. Future studies are warranted to investigate a wider array of features.

## Supplementary Information

Below is the link to the electronic supplementary material.

Supplementary file1 (DOCX 27 KB)

## Data Availability

The study dataset is available upon reasonable request.

## References

[1] Brown GW, Birley JL, Wing JK (1972) Influence of family life on the course of schizophrenic disorders: a replication. Br J Psychiatry 121(562):241–258. 10.1192/bjp.121.3.241 5073778 10.1192/bjp.121.3.241

[2] Vaughn CE, Leff JP (1976) The influence of family and social factors on the course of psychiatric illness. a comparison of schizophrenic and depressed neurotic patients. Br J Psychiatry 129:125–137. 10.1192/bjp.129.2.125 963348 10.1192/bjp.129.2.125

[3] Alvarez-Jimenez M, Priede A, Hetrick SE, Bendall S, Killackey E, Parker AG, McGorry PD, Gleeson JF (2012) Risk factors for relapse following treatment for first episode psychosis: a systematic review and meta-analysis of longitudinal studies. Schizophr Res 139(1–3):116–128. 10.1016/j.schres.2012.05.007 22658527 10.1016/j.schres.2012.05.007

[4] Vaughn C, Leff J (1976) The measurement of expressed emotion in the families of psychiatric patients. Br J Soc Clin Psychol 15(2):157–165. 10.1111/j.2044-8260.1976.tb00021.x 938822 10.1111/j.2044-8260.1976.tb00021.x

[5] Wearden AJ, Tarrier N, Barrowclough C, Zastowny TR, Rahill AA (2000) A review of expressed emotion research in health care. Clin Psychol Rev 20(5):633–666. 10.1016/s0272-7358(99)00008-2 10860170 10.1016/s0272-7358(99)00008-2

[6] Hooley JM, Parker HA (2006) Measuring expressed emotion: an evaluation of the shortcuts. J Fam Psychol 20(3):386–396. 10.1037/0893-3200.20.3.386 16937995 10.1037/0893-3200.20.3.386

[7] Van Humbeeck G, Van Audenhove C, De Hert M, Pieters G, Storms G (2002) Expressed emotion: a review of assessment instruments. Clin Psychol Rev 22(3):321–341. 10.1016/S0272-7358(01)00098-8 10.1016/s0272-7358(01)00098-8 17201189

[8] Kreisman DE, Simmens SJ, Joy VD (1979) Rejecting the patient: preliminary validation of a self-report scale. Schizophr Bull 5(2):220–222. 10.1093/schbul/5.2.220 462140 10.1093/schbul/5.2.220

[9] Kavanagh DJ, O’Halloran P, Manicavasagar V, Clark D, Piatkowska O, Tennant C, Rosen A (1997) The family attitude scale: reliability and validity of a new scale for measuring the emotional climate of families. Psychiatry Res 70(3):185–195. 10.1016/s0165-1781(97)00033-4 9211580 10.1016/s0165-1781(97)00033-4

[10] Wiedemann G, Rayki O, Feinstein E, Hahlweg K (2002) The Family Questionnaire: development and validation of a new self-report scale for assessing expressed emotion. Psychiatry Res 109(3):265–279. 10.1016/s0165-1781(02)00023-9 11959363 10.1016/s0165-1781(02)00023-9

[11] Butzlaff RL, Hooley JM (1998) Expressed emotion and psychiatric relapse: a meta-analysis. Arch Gen Psychiatry 55(6):547–552. 10.1001/archpsyc.55.6.547 9633674 10.1001/archpsyc.55.6.547

[12] Ma CF, Chan SKW, Chung YL, Ng SM, Hui CLM, Suen YN, Chen EYH (2021) The predictive power of expressed emotion and its components in relapse of schizophrenia: a meta-analysis and meta-regression. Psychol Med 51(3):365–375. 10.1017/s0033291721000209 33568244 10.1017/S0033291721000209

[13] Butler R, Berry K, Varese F, Bucci S (2019) Are family warmth and positive remarks related to outcomes in psychosis? Syst Rev Psychol Med 49(8):1250–1265. 10.1017/s0033291718003768 10.1017/S0033291718003768 30569884

[14] Berry K, Barrowclough C, Haddock G (2011) The role of expressed emotion in relationships between psychiatric staff and people with a diagnosis of psychosis: a review of the literature. Schizophr Bull 37(5):958–972. 10.1093/schbul/sbp162 20056685 10.1093/schbul/sbp162 PMC3160217

[15] Katsuki F, Goto M, Someya T (2005) A study of emotional attitude of psychiatric nurses: reliability and validity of the nurse attitude scale. Int J Ment Health Nurs 14(4):265–270. 10.1111/j.1440-0979.2005.00391.x 16296994 10.1111/j.1440-0979.2005.00391.x

[16] Snyder KS, Wallace CJ, Moe K, Liberman RP (1994) Expressed emotion by residential care operators and residents’ symptoms and quality of life. Psychiatr Serv 45(11):1141–1143. 10.1176/ps.45.11.1141 10.1176/ps.45.11.1141 7835864

[17] Tattan T, Tarrier N (2000) The expressed emotion of case managers of the seriously mentally ill: the influence of expressed emotion on clinical outcomes. Psychol Med 30(1):195–204. 10.1017/s0033291799001579 10722190 10.1017/s0033291799001579

[18] Ball RA, Moore E, Kuipers L (1992) Expressed emotion in community care staff: a comparison of patient outcome in a 9-month follow-up of two hostels. Soc Psychiatry Psychiatr Epidemiol 27:35–39 1557680 10.1007/BF00788954

[19] Solomon P, Alexander L, Uhl S (2010) The relationship of case managers’ expressed emotion to clients’ outcomes. Soc Psychiatry Psychiatr Epidemiol 45(2):165–174. 10.1007/s00127-009-0051-3 19370297 10.1007/s00127-009-0051-3

[20] Amaresha AC, Venkatasubramanian G (2012) Expressed emotion in schizophrenia: an overview. Indian J Psychol Med 34(1):12–20. 10.4103/0253-7176.96149 22661801 10.4103/0253-7176.96149 PMC3361836

[21] Alvarez-Jiménez M, Gleeson JF, Cotton SM, Wade D, Crisp K, Yap MB, McGorry PD (2010) Differential predictors of critical comments and emotional over-involvement in first-episode psychosis. Psychol Med 40(1):63–72. 10.1017/s0033291708004765 19079825 10.1017/S0033291708004765

[22] Tompson MC, Goldstein MJ, Lebell MB, Mintz LI, Marder SR, Mintz J (1995) Schizophrenic patients’ perceptions of their relatives’ attitudes. Psychiatry Res 57(2):155–167. 10.1016/0165-1781(95)02598-q 7480382 10.1016/0165-1781(95)02598-q

[23] Lebell MB, Marder SR, Mintz J, Mintz LI, Tompson M, Wirshing W, Johnston-Cronk K, McKenzie J (1993) Patients’ perceptions of family emotional climate and outcome in schizophrenia. Br J Psychiatry 162:751–754. 10.1192/bjp.162.6.751 8330106 10.1192/bjp.162.6.751

[24] Barrowclough C, Haddock G, Lowens I, Connor A, Pidliswyj J, Tracey N (2001) Staff expressed emotion and causal attributions for client problems on a low security unit: an exploratory study. Schizophr Bull 27(3):517–526. 10.1093/oxfordjournals.schbul.a006892 11596852 10.1093/oxfordjournals.schbul.a006892

[25] Van Humbeeck G, Van Audenhove C, Pieters G, De Hert M, Storms G, Vertommen H, Peuskens J, Heyrman J (2001) Expressed emotion in staff-patient relationships: the professionals’ and residents’ perspectives. Soc Psychiatry Psychiatr Epidemiol 36(10):486–492. 10.1007/s001270170013 11768846 10.1007/s001270170013

[26] Davis JA, Goldstein MJ, Nuechterlein KH (1996) Gender differences in family attitudes about schizophrenia. Psychol Med 26(4):689–696. 10.1017/s0033291700037703 8817703 10.1017/s0033291700037703

[27] Zanetti ACG, Vedana KGG, Pereira CCM, de Azevedo Marques JM, da Silva AHS, Martin IdS, Dantas RAS, de Souza J, Galera SAF, Gherardi-Donato ECdS (2019) Expressed emotion and socio-demographic and clinical factors in families of Brazilian patients with schizophrenia. Int J Soc Psychiatry 65(1):56–63. 10.1177/0020764018815207 30488742 10.1177/0020764018815207

[28] Bentsen H, Notland TH, Boye B, Munkvold OG, Bjørge H, Lersbryggen AB, Uren G, Oskarsson KH, Berg-Larsen R, Lingjaerde O, Malt UF (1998) Criticism and hostility in relatives of patients with schizophrenia or related psychoses: demographic and clinical predictors. Acta Psychiatr Scand 97(1):76–85. 10.1111/j.1600-0447.1998.tb09967.x 9504708 10.1111/j.1600-0447.1998.tb09967.x

[29] Van Humbeeck G, Van Audenhove C, Pieters G, De Hert M, Storms G, Vertommen H, Peuskens J, Heyrman J (2002) Expressed emotion in the client-professional caregiver dyad: are symptoms, coping strategies and personality related? Soc Psychiatry Psychiatr Epidemiol 37(8):364–371. 10.1007/s00127-002-0565-4 12195543 10.1007/s00127-002-0565-4

[30] Koutra K, Triliva S, Roumeliotaki T, Lionis C, Vgontzas AN (2015) Identifying the socio-demographic and clinical determinants of family functioning in Greek patients with psychosis. Int J Soc Psychiatry 61(3):251–264. 10.1177/0020764014540151 24972747 10.1177/0020764014540151

[31] van Os J, Marcelis M, Germeys I, Graven S, Delespaul P (2001) High expressed emotion: marker for a caring family? Compr Psychiatry 42(6):504–507. 10.1053/comp.2001.27899 11704944 10.1053/comp.2001.27899

[32] Karanci AN, Inandilar H (2002) Predictors of components of expressed emotion in major caregivers of Turkish patients with schizophrenia. Soc Psychiatry Psychiatr Epidemiol 37(2):80–88. 10.1007/s127-002-8219-1 11931092 10.1007/s127-002-8219-1

[33] Moore E, Ball RA, Kuipers L (1992) Expressed emotion in staff working with the long-term adult mentally ill. Br J Psychiatry 161:802–808. 10.1192/bjp.161.6.802 1483166 10.1192/bjp.161.6.802

[34] Oliver N, Kuipers E (1996) Stress and its relationship to expressed emotion in community mental health workers. Int J Soc Psychiatry 42(2):150–159. 10.1177/002076409604200209 8811399 10.1177/002076409604200209

[35] Hall MJ, Docherty NM (2000) Parent coping styles and schizophrenic patient behavior as predictors of expressed emotion. Fam Process 39(4):435–444. 10.1111/j.1545-5300.2000.39404.x 11143597 10.1111/j.1545-5300.2000.39404.x

[36] Bentsen H, Boye B, Munkvold OG, Notland TH, Lersbryggen AB, Oskarsson KH, Ulstein I, Uren G, Bjørge H, Berg-Larsen R, Lingjaerde O, Malt UF (1996) Emotional overinvolvement in parents of patients with schizophrenia or related psychosis: demographic and clinical predictors. Br J Psychiatry 169(5):622–630. 10.1192/bjp.169.5.622 8932893 10.1192/bjp.169.5.622

[37] Levy E, Shefler G, Loewenthal U, Umansky R, Bar G, Heresco-Levy U (2005) Characteristics of schizophrenia residents and staff rejection in community mental health hostels. Isr J Psychiatry Relat Sci 42(1):23–32 16134404

[38] Barrowclough C, Johnston M, Tarrier N (1994) Attributions, expressed emotion, and patient relapse: an attributional model of relatives’ response to schizophrenic illness. Behav Ther 25(1):67–88. 10.1016/S0005-7894(05)80146-7

[39] Brewin CR, MacCarthy B, Duda K, Vaughn CE (1991) Attribution and expressed emotion in the relatives of patients with schizophrenia. J Abnorm Psychol 100(4):546–554. 10.1037/0021-843x.100.4.546 1757668 10.1037//0021-843x.100.4.546

[40] Hinojosa-Marqués L, Domínguez-Martínez T, Kwapil TR, Barrantes-Vidal N (2020) Predictors of criticism and emotional over-involvement in relatives of early psychosis patients. PLoS ONE 15(6):e0234325. 10.1371/journal.pone.0234325 32542020 10.1371/journal.pone.0234325 PMC7295211

[41] Fahrer J, Brill N, Dobener LM, Asbrand J, Christiansen H (2021) Expressed emotion in the family: a meta-analytic review of expressed emotion as a mechanism of the transgenerational transmission of mental disorders. Front Psychiatry 12:721796. 10.3389/fpsyt.2021.721796 35177995 10.3389/fpsyt.2021.721796 PMC8846301

[42] da Silva AHS, de Souza TL, de Azevedo-Marques JM, Shuhama R, Del-Ben CM, Galera SAF, da Silva Gherardi-Donato EC, Vedana KGG, Zanetti ACG (2020) Predictors of expressed emotion in first episode psychosis. Issues Ment Health Nurs 41(10):908–915. 10.1080/01612840.2020.1749916 32568611 10.1080/01612840.2020.1749916

[43] Wei Y, Peng Y, Li Y, Song L, Ju K, Xi J (2022) Caregivers’ burden and schizophrenia patients’ quality of life: Sequential mediating effects of expressed emotion and perceived expressed emotion. Front Psychiatry. 10.3389/fpsyt.2022.961691 36090381 10.3389/fpsyt.2022.961691 PMC9454947

[44] Katsuki F, Fukui S, Niekawa N, Oshima I, Setoya N, Ninomiya S, Moriyama A, Uchino T, Ito J, Tsukada K (2008) Development of the Nurse Attitude Scale short form: factor analysis in a large sample of Japanese psychiatric clinical staff. Psychiatry Clin Neurosci 62(3):349–351. 10.1111/j.1440-1819.2008.01803.x 18588597 10.1111/j.1440-1819.2008.01803.x

[45] Overall JE, Gorham DR (1962) The brief psychiatric rating scale. Psychol Rep 10(3):799–812. 10.2466/pr0.1962.10.3.799

[46] Burger GK, Calsyn RJ, Morse GA, Klinkenberg WD, Trusty ML (1997) Factor structure of the expanded brief psychiatric rating scale. J Clin Psychol 53(5):451–454. 10.1002/(sici)1097-4679(199708)53:5%3c451::aid-jclp5%3e3.0.co;2-q 9257222 10.1002/(sici)1097-4679(199708)53:5<451::aid-jclp5>3.0.co;2-q

[47] Paneras A, Crawford J (2004) The use of the brief psychiatric rating scale in Greek psychiatric patients. Sci Ann Rep 2:183–201

[48] Hooley JM, Teasdale JD (1989) Predictors of relapse in unipolar depressives: expressed emotion, marital distress, and perceived criticism. J Abnorm Psychol 98(3):229–235. 10.1037/0021-843x.98.3.229 2768657 10.1037//0021-843x.98.3.229

[49] Medina-Pradas C, Navarro JB, Pousa E, Montero MI, Obiols JE (2013) Expressed and perceived criticism, family warmth, and symptoms in schizophrenia. Span J Psychol 16:E45. 10.1017/sjp.2013.25 23866241 10.1017/sjp.2013.25

[50] Onwumere J, Kuipers E, Bebbington P, Dunn G, Freeman D, Fowler D, Garety P (2009) Patient perceptions of caregiver criticism in psychosis: links with patient and caregiver functioning. J Nerv Ment Dis 197(2):85–91. 10.1097/NMD.0b013e3181960e57 19214042 10.1097/NMD.0b013e3181960e57

[51] Maslach C, Jackson SE (1981) The measurement of experienced burnout. J Organ Behav 2(2):99–113. 10.1002/job.4030020205

[52] Maslach C, Jackson SE, Leiter MP (1996) Maslach Burnout Inventory Manual. CPP Mountain View, CA

[53] Anagnostopoulos F, Papadatou D (1992) Factorial composition and internal consistency of the Greek version of the Maslach Burnout Inventory in a sample of nurses. Psychol Rep 5(3):183–202

[54] Madianos M, Economou M, Dafni O, Koukia E, Palli A, Rogakou E (2004) Family disruption, economic hardship and psychological distress in schizophrenia: can they be measured? Eur Psychiatry 19(7):408–414. 10.1016/j.eurpsy.2004.06.028 15504647 10.1016/j.eurpsy.2004.06.028

[55] Magaña AB, Goldstein JM, Karno M, Miklowitz DJ, Jenkins J, Falloon IR (1986) A brief method for assessing expressed emotion in relatives of psychiatric patients. Psychiatry Res 17(3):203–212. 10.1016/0165-1781(86)90049-1 3704028 10.1016/0165-1781(86)90049-1

[56] Mazza C, Formica F, Ferracuti S, Ricci E, Colasanti M, Biondi S, Di Domenico A, Roma P (2022) High expressed emotion (HEE), assessed using the five-minute speech sample (FMSS), as a predictor of psychiatric relapse in patients with schizophrenia and major depressive disorder: a meta-analysis and meta-regression. J Clin Med. 10.3390/jcm11216533 36362760 10.3390/jcm11216533 PMC9658267

[57] Meng X-l, Rosenthal R, Rubin DB (1992) Comparing correlated correlation coefficients. Psychol Bull 111(1):172–175. 10.1037/0033-2909.111.1.172

[58] Leucht S, Kane JM, Kissling W, Hamann J, Etschel E, Engel R (2005) Clinical implications of brief psychiatric rating scale scores. Br J Psychiatry 187:366–371. 10.1192/bjp.187.4.366 16199797 10.1192/bjp.187.4.366

[59] Guy W (1976) ECDEU assessment manual for psychopharmacology. Department of Health Education, Welfare

[60] Kavanagh DJ (1992) Recent developments in expressed emotion and schizophrenia. Br J Psychiatry 160:601–620. 10.1192/bjp.160.5.601 1591571 10.1192/bjp.160.5.601

[61] Francis A, Papageorgiou P (2004) Expressed emotion in Greek versus Anglo-Saxon families of individuals with schizophrenia. Aust Psychol 39(2):172–177. 10.1080/00050060410001701898

[62] Singh SP, Harley K, Suhail K (2013) Cultural specificity of emotional overinvolvement: a systematic review. Schizophr Bull 39(2):449–463. 10.1093/schbul/sbr170 22190078 10.1093/schbul/sbr170 PMC3576159

[63] Tarrier N, Barrowclough C, Andrews B, Gregg L (2004) Risk of non-fatal suicide ideation and behaviour in recent onset schizophrenia–the influence of clinical, social, self-esteem and demographic factors. Soc Psychiatry Psychiatr Epidemiol 39(11):927–937. 10.1007/s00127-004-0828-3 15549247 10.1007/s00127-004-0828-3

[64] Falloon IR (2003) Family interventions for mental disorders: efficacy and effectiveness. World Psychiatry 2(1):20–28 16946881 PMC1525058

[65] Kim S-H, Park S (2023) Effectiveness of family interventions for patients with schizophrenia: a systematic review and meta-analysis. Int J Ment Health Nurs. 10.1111/inm.13198 37553813 10.1111/inm.13198

[66] Willetts LE, Leff J (1997) Expressed emotion and schizophrenia: the efficacy of a staff training programme. J Adv Nurs 26(6):1125–1133. 10.1111/j.1365-2648.1997.tb00804.x 9429962 10.1111/j.1365-2648.1997.tb00804.x

[67] Endley L, Berry K (2011) Increasing awareness of expressed emotion in schizophrenia: an evaluation of a staff training session. J Psychiatr Ment Health Nurs 18(3):277–280. 10.1111/j.1365-2850.2010.01683.x 21395920 10.1111/j.1365-2850.2010.01683.x

[68] Chadda RK, Singh TB, Ganguly KK (2007) Caregiver burden and coping. Soc Psychiatry Psychiatr Epidemiol 42(11):923–930. 10.1007/s00127-007-0242-8 17700975 10.1007/s00127-007-0242-8

[69] O’Connor K, Muller Neff D, Pitman S (2018) Burnout in mental health professionals: a systematic review and meta-analysis of prevalence and determinants. Eur Psychiatry 53:74–99. 10.1016/j.eurpsy.2018.06.003 29957371 10.1016/j.eurpsy.2018.06.003

