# Supplementary material for: Differential correlates of criticism versus emotional overinvolvement towards patients with schizophrenia living in halfway houses or with their families
Source: Soc Psychiatry Psychiatr Epidemiol. 2024 Jan 31;59(10):1761–73. doi: 10.1007/s00127-023-02609-7 (PMC11464640; doi:10.1007/s00127-023-02609-7)
Supplement: Supplementary file 1 — Supplementary file1 (DOCX 27 KB) [file 127_2023_2609_MOESM1_ESM.docx]

**Supplementary Methods**

**FMSS scoring**

Criticism and Emotional Overinvolvement (EOI), the two dimensions of EE, are rated based on conditions grouped in four categories: initial statement, quality of relationship, criticism, and EOI. The first three are used to rate Criticism and the last to rate EOI. Criticism and EOI are finally coded as high, borderline and low. Borderline can also be grouped with low to provide a dichotomous rating (high vs borderline/low).

**High Criticism** is coded if the rater (a) makes a negative initial statement about the patient; the initial statement can be rated as negative, neutral or positive; or (b) describes the relationship with the patient as negative; statements about the quality of the relationship are individually rated as weakly or strongly negative (-1 or -2) or positive (+1 or +2) and then summed up giving the overall relationship rating (negative, neutral or positive); or (c) makes at least one critical (i.e. strongly disapproving) comment, rated on the basis of content and voice tone. **Borderline Criticism** is coded if the rater only makes dissatisfaction comments.

**High EOI** is coded if the rater (a) reports or displays overprotective or self-sacrificing behavior or lack of objectivity towards the patient; or (b) exhibits intense emotional display during the interview; or (c) any two of the following are true: (1) describes the past or a minor characteristic of the patient in excessive detail, (2) makes at least one statement of positive attitude towards the patient, (3) makes five or more positive comments about the patient. **Borderline EOI** is coded if only one of the aforementioned (c) conditions is true.

The final seven EE categories arise from combining Criticism and EOI categories: high critical, high EOI, high critical+EOI, borderline critical, borderline EOI, borderline critical+EOI, low critical+EOI (or low EE). If borderline is grouped with low, one will have four categories: high critical, high EOI, high critical+EOI, borderline/low critical+EOI. Finally, one can have two major categories of high EE (critical and/or EOI) and borderline/low EE (critical and/or EOI).

Suppl. Table 1. Questionnaire reliability for patients (N=80), Nurses (N=22) and Parents (N=56).

|  | **No of Questions** | **Range of Score** | **Cronbach’s α** |
| --- | --- | --- | --- |
| **Patients** |  |  |  |
| **BPRS Thinking Disorder** | 4 | 4-28 | 0.728 |
| **BPRS Withdrawal** | 4 | 4-28 | 0.703 |
| **BPRS Anxiety/ Depression** | 4 | 4-28 | 0.726 |
| **BPRS Hostility/ Suspicion** | 3 | 3-21 | 0.722 |
| **BPRS Activity** | 3 | 3-21 | 0.712 |
| **BPRS Total** | 18 | 18-126 | 0.783 |
| **Perceived criticism** | 1 | 1-10 | - |
|  |  |  |  |
| **Nurses** |  |  |  |
| **MBI Emotional Exhaustion** | 9 | 0-54 | 0.861 |
| **MBI Personal Achievements** | 8 | 0-48 | 0.754 |
| **MBI Depersonalization** | 5 | 0-30 | 0.703 |
|  |  |  |  |
| **Parents** |  |  |  |
| **FBS Financial Burden** | 5 | 0-10 | 0.701 |
| **FBS Impact on Daily Activities/ Social Life** | 8 | 0-16 | 0.796 |
| **FBS Aggressive Behavior** | 4 | 0-8 | 0.729 |
| **FBS Impact on Health** | 6 | 0-12 | 0.784 |
| **FBS Total** | 23 | 0-46 | 0.843 |

BPRS, Brief Psychiatric Rating Scale; FBS, Family Burden Scale; MBI, Maslach Burnout Inventory

Suppl. Table 2. Rater samples description: Staff Nurses (N=22) and Parents (N=56).

| **Nurses (N=22)** | |  | **Parents (N=56)** | |  |
| --- | --- | --- | --- | --- | --- |
|  | **N(%)** |  |  | **N(%)** | **p-value** |
| **Gender** (Male) | 6(27.3%) |  | **Gender** (Male) | 24(42.9%) | 0.203^a^ |
| **Education** |  |  | **Education** |  | **0.001**^b^ |
| Primary School | 0(0.0%) |  | Primary School | 20(35.7%) |  |
| High School | 8(36.4%) |  | High School | 19(33.9%) |  |
| University or higher | 14(63.6%) |  | University or higher | 17(30.4%) |  |
| **Work Experience** |  |  | **Employment** |  |  |
| 0-5 years | 3(13.6%) |  | Employed | 8(14.3%) |  |
| 5-11 years | 6(27.3%) |  | Unemployed | 11(19.6%) |  |
| >11 years | 13(59.1%) |  | Retired | 37(66.1%) |  |
| **Family Status** |  |  | **Psychiatric History** |  |  |
| Single | 8(36.4%) |  | No | 49(87.5%) |  |
| Married | 14(63.6%) |  | Yes | 7(12.5%) |  |
|  |  |  |  |  |  |
|  | **Mean±SD** |  |  | **Mean±SD** |  |
| **Age (years)** | 40.0±7.2 |  | **Age (years)** | 68.0±8.6 | **<0.001**^c^ |
|  |  |  |  |  |  |
| **MBI Emotional Exhaustion** | 12.4±8.5 |  | **FBS Financial Burden** | 4.1±2.2 |  |
| **MBI Personal Achievements** | 37.3±5.3 |  | **FBS Impact on Daily Activities/ Social Life** | 5.8±4.4 |  |
| **MBI Depersonalization** | 3.5±2.9 |  | **FBS Aggressive Behavior** | 1.0±1.4 |  |
|  |  |  | **FBS Impact on Health** | 5.2±3.4 |  |
|  |  |  | **FBS Total** | 16.1±8.6 |  |
|  |  |  |  |  |  |

N(%) or mean±SD are presented. Chi-square,^a^ Fisher’s exact^b^ or t-test^c^ were used as appropriate.

FBS, Family Burden Scale; MBI, Maslach Burnout Inventory

Bold p<0.05
